# The roles of replication-transcription conflict in mutagenesis and evolution of genome organization

**DOI:** 10.1371/journal.pgen.1008987

**Published:** 2020-08-27

**Authors:** Jeremy W. Schroeder, T. Sabari Sankar, Jue D. Wang, Lyle A. Simmons

**Affiliations:** 1 Department of Bacteriology, University of Wisconsin-Madison, Madison, Wisconsin, United States of America; 2 School of Biology, Indian Institute of Science Education and Research Thiruvananthapuram, Thiruvananthapuram, Kerala, India; 3 Department of Molecular, Cellular, and Developmental Biology, University of Michigan, Ann Arbor, Michigan, United States of America; Washington University in St. Louis, UNITED STATES

## Abstract

Replication-transcription conflicts promote mutagenesis and give rise to evolutionary signatures, with fundamental importance to genome stability ranging from bacteria to metastatic cancer cells. This review focuses on the interplay between replication-transcription conflicts and the evolution of gene directionality. In most bacteria, the majority of genes are encoded on the leading strand of replication such that their transcription is co-directional with the direction of DNA replication fork movement. This gene strand bias arises primarily due to negative selection against deleterious consequences of head-on replication-transcription conflict. However, many genes remain head-on. Can head-on orientation provide some benefit? We combine insights from both mechanistic and evolutionary studies, review published work, and analyze gene expression data to evaluate an emerging model that head-on genes are temporal targets for adaptive mutagenesis during stress. We highlight the alternative explanation that genes in the head-on orientation may simply be the result of genomic inversions and relaxed selection acting on nonessential genes. We seek to clarify how the mechanisms of replication-transcription conflict, in concert with other mutagenic mechanisms, balanced by natural selection, have shaped bacterial genome evolution.

## Introduction

Studies of mutagenesis in bacteria have revealed conserved biological processes influencing mutation rate across all domains of life and identified sources of bacterial evolution against host defense, peer competition, and antibiotic exposure. Historical studies of bacterial mutations by Luria and Delbrück led to a conclusive demonstration that spontaneous mutations generate the genetic diversity to allow survival of subsequent selective pressure [1]. Mutations do not arise uniformly across the genome, because multiple mechanisms for mutagenesis exist and the effect of each mechanism depends on the genomic context of a given locus [2]. Replication-transcription conflict is one mutagenic mechanism that is particularly influenced by genomic context. Replication-transcription conflict is common to prokaryotic and eukaryotic systems (for reviews, see [3–6]). Insertions and deletions within genes, a key mutation signature of replication-transcription conflict in bacteria, are also found in cancer cells [7–9], suggesting that the mechanisms by which conflicts generate mutations may also be conserved.

**Fig 1:**
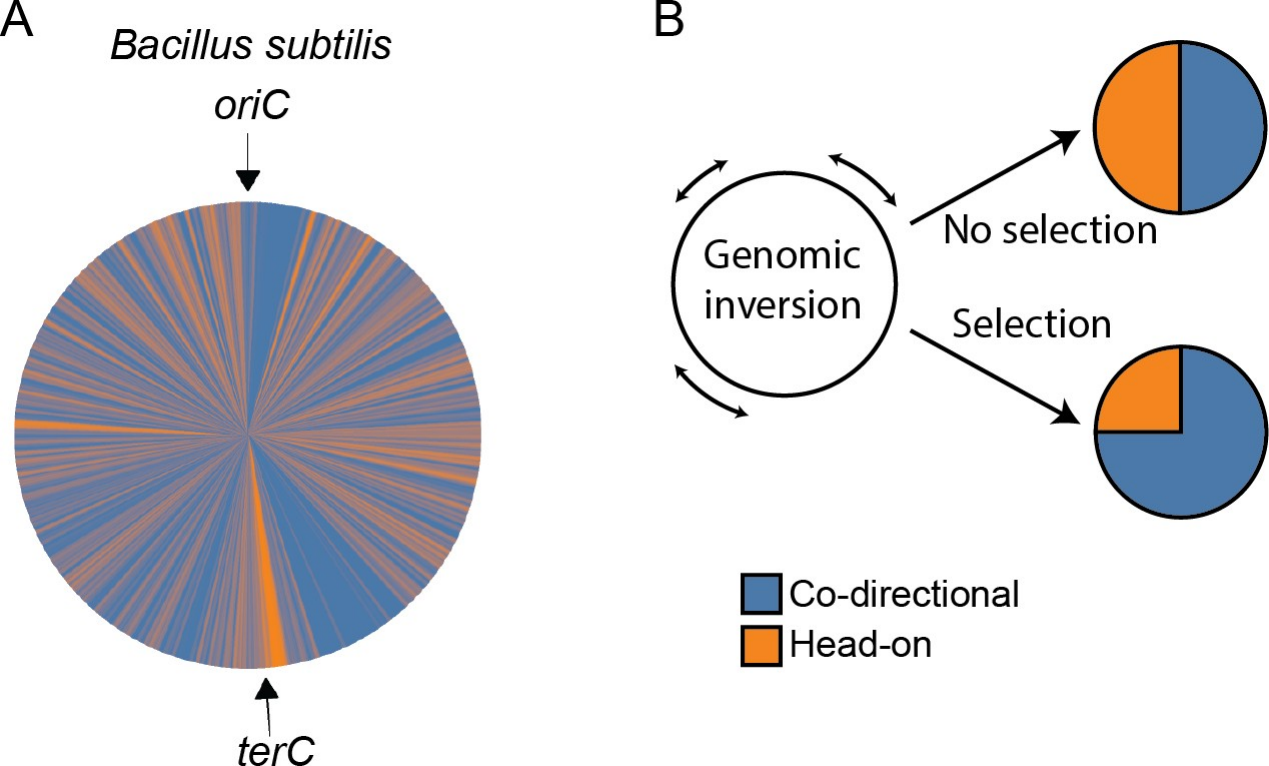
Genome-wide bias against head-on genes reflects negative selection. (A) The *B*. *subtilis* genome is represented to scale as a circle filled with blue or orange to represent loci of the genome containing co-directional and head-on genes, respectively. The origin (*oriC*) and terminus (*terC*) of replication are labelled. Strain PY79 is shown but the basic genome organization is conserved among *B*. *subtilis* strains. (B) Schematic of random inversions of genomic loci and evolutionary outcomes depending on selection. In the absence of selection against head-on genes, genomic inversions over time would eventually result in a 50:50 distribution of head-on:co-directional genes. In the presence of selection against head-on genes, gene content is biased in favor of co-directional genes. Genes that remain head-on are typically those for which there is less selection against the head-on orientation.

Recently, the field of replication-transcription conflict has generated both excitement and contradictions. There are new developments in the contribution of conflicting interplay between replication-transcription machineries to genome evolution and genome organization. In most bacterial species, the majority of genes are encoded in the leading strand of replication such that their transcription is co-directional with DNA replication fork movement. The extent of gene strand bias differs across bacteria, with *Escherichia coli*, *Bacillus subtilis*, and *Thermoanaerobacter tengcongensis* having 55%, 75%, and 87% of genes co-directional, respectively (Fig 1A and [5]). Gene strand bias is the result of purifying selection against head-on genes, which are targets for mutagenesis and impede DNA replication when they are highly expressed (Fig 1B and [7,10–12]). Experimental evidence demonstrates that deleterious consequences of inverting co-directionally oriented genes to be head-on include slowed replication fork progression, replication arrest, and DNA double-strand breaks/ends [7,10,13,14].

Given the deleterious consequences of head-on conflict, it is natural to wonder how bacterial genomes have not evolved to have all genes in the co-directional orientation. After all, such an arrangement of genes should increase genome stability and fitness. Is there a driving force for some genes to be oriented head-on? One possibility is that head-on orientation of stress-induced genes is favorable. Merrikh and colleagues proposed that stress-induced genes are preferentially positioned in the head-on orientation to promote replication-transcription conflict during stress, which in turn would promote adaptive mutagenesis of stress-induced genes [15–19]. Alternatively, the prevalence of stress-induced genes in the head-on orientation may simply be the result of two well-established phenomena: the occurrence of genomic inversions and selection against head-on genes. This alternative model is supported by recent work documenting the types of mutations generated by head-on conflict, the mechanisms of mutagenesis in head-on genes, and the evolutionary forces driving strand-biased gene distribution [7,20–23]. In this review, we present the evidence raised to support each view. We first briefly review evolutionary work pertaining to head-on genes, then discuss the mechanisms of mutagenesis of head-on genes, both dependent and independent of conflict, and finally examine stress-induced genes specifically. We hope to clarify what has been learned about replication-transcription conflict as it pertains to mutagenesis and evolution of head-on genes and strand-biased gene distribution.

## An evolutionary explanation for the existence of head-on genes

Merrikh and colleagues proposed an adaptive hypothesis to explain the occurrence of genes in the head-on orientation [17,19]. Using comparative genomics, they presented several lines of evidence they propose support positive selection for head-on oriented genes. First, estimating nucleotide substitutions of several genes, they showed that nonsynonymous substitutions (dN) were higher in head-on compared to co-directional genes, while synonymous substitutions (dS) showed no difference. Therefore, the ratio of dN to dS, which is an indicator of selective pressure on protein-coding genes, was higher for head-on genes [17,19]. Second, dN/dS ratios that are significantly greater than one provide evidence that a gene is under positive selection [24], and the authors showed that genes with dN/dS modestly above one from multiple bacterial species were slightly more enriched in head-on genes compared to co-directional genes [17]. Finally, they proposed that convergent amino acid substitutions, a key criterion of adaptive evolution, occurred more frequently in head-on genes [19]. Based on these observations, Merrikh and colleagues proposed that head-on genes experience positive selection for increased mutation rate caused by head-on conflicts.

Although the adaptive hypothesis is intriguing, the notion is contradicted based on the theory of mutation-selection balance [20,23]. First, the increased dN observed for lagging strand-encoded genes can be explained by either adaptive evolution or relaxed purifying selection. In this case, purifying selection is the selection against deleterious mutations. Co-directional genes are enriched with essential genes and housekeeping genes, and are therefore subject to stronger purifying selection than head-on genes. Nonsynonymous substitutions in co-directional genes are selected against, causing lower dN and dN/dS in co-directional genes. The finding that the mean dN/dS ratio of head-on genes is slightly higher than that of co-directional genes [17,19], while remaining well below one, provides strong support that head-on genes are under relaxed purifying selection compared with co-directional genes. Second, although the percentage of genes with dN/dS greater than one, i.e., under positive selection, is higher among genes in the head-on orientation, in absolute numbers, more co-directional genes counted by Merrikh and Merrikh have dN/dS greater than one (289 of 15,627 total co-directional genes versus 234 of 9,757 total head-on genes) [17]. Thus, on a gene-by-gene basis there is little evidence to suggest that genes with dN/dS greater than one receive additional adaptive benefits if they are head-on. Therefore, there remains poor evidence to suggest positive selection for head-on genes. Finally, contrary to the claims of Merrikh and colleagues [19], Chen and Zhang [20] show that convergent substitution, a key criterion of adaptive evolution [25], is not observed to be higher in head-on genes.

Instead of the head-on orientation being positively selected, gene strand bias can represent the equilibrium distribution of head-on and co-directional genes that arises due to a balance of random genetic inversion and purifying selection against head-on genes (Fig 1B and [20]). Therefore, despite negative selection against head-on genes in general, genes that remain head-on are those whose head-on conflict is either less costly to bacterial fitness, or that have not yet been purified from the genome. In agreement with this theory, the strength of selection against the head-on orientation depends on the type of gene in which conflict occurs. For example, inverting the ribosomal RNA operons to the head-on orientation has extremely deleterious consequences because they are highly expressed essential genes, and their head-on transcription disrupts replication fork progression [4,10,11,26]. Therefore, the extreme consequences of head-on conflict at inverted ribosomal RNA operons explain how all ribosomal RNA operons remain oriented co-directional to replication [27]. Mutation-selection balance may also explain differences in gene strand bias between different bacteria. Inverting genes from co-directional to head-on results in a much stronger fitness defect in *B*. *subtilis* [10] compared with similar inversions in *E*. *coli* [28,29]. Accordingly, *B*. *subtilis* has higher bias for co-directional genes (75% co-directional) than *E*. *coli* (55% co-directional) [5]. Thus, natural selection favors genes to be co-directionally transcribed [6], but due to unavoidable inversion events, the presence of head-on genes is inevitable.

Are there sufficient inversion events to support the mutation-selection balance model for gene strand bias? Bacteria often contain a circular chromosome with a single origin of replication and two replication forks, each replicating half a chromosome (replichore). Any inversion event contained within a replichore will change the relative direction of replication-transcription conflict for all genes contained within the inversion. Inversions within a replichore would rarely be detected at the population level, and this is likely due to the fitness cost of having many genes oriented head-on [10,30,31]. Bioinformatic studies in many bacteria reveal that symmetric inversions around the origin of replication frequently occur [32,33]. This further suggests that nonsymmetric inversions may be more common than currently realized, but only symmetric inversions are pervasively retained through evolution due to their low fitness cost. In studies selecting for inversion within a gene bearing 12- or 23-bp regions of inverted homology, Miller and co-workers estimated inversion rates to be 4 × 10^−9^ to 1 × 10^−7^ per generation [34,35]. Inversion rates will depend on many factors, including the length of homology at the inversion break-points [36]. For comparison, the rate of base-pair substitution in bacteria varies around 3 × 10^−10^ per nucleotide replicated per generation, depending on the type of substitution and sequence context [22,37]. Interestingly, both the Zhang group and the Merrikh group, although disagreeing in the numbers, provide evidence for many inversion events resulting in co-directional to head-on changes of gene orientation [17,21]. In summary, considering the potential rate of chromosomal inversions, in both methodological and theoretical terms the mutation-selection balance model best explains the existence of head-on genes [20,21,23].

## Mutation signatures of head-on conflict: Deleterious or beneficial?

Various reporters have been employed to study the mutagenic consequences of replication-transcription conflicts. Mutation assays based on resistance to rifampin or trimethoprim [7,10], and reversion of auxotrophic mutants to prototrophy [19], have all shown that mutation reporter genes in the head-on orientation have modestly higher mutation rates than their co-directional counterpart in *B*. *subtilis*. These similar results have been interpreted differently. The Merrikh group interpreted the increased rate of reversion of auxotrophic mutants to prototrophy to support their hypothesis favoring conflict-induced mutations driving positive selection for head-on genes [18,19]. However, their assays begin with a nonfunctional mutant gene and select for specific reversions to restore a functional state. During natural evolution, head-on genes do not begin with a nonfunctional state wherein the only direction for fitness to go is up. Rather, under more natural conditions, mutagenesis comes with many risks to gene fitness. In addition, reversion assays only select for specific nonsynonymous base substitutions at few positions, making them poorly suited to study the impact of conflicts on mutagenesis. In contrast, forward mutation assays select for all loss-of-function mutations in a gene and are thus more helpful in revealing the multiple types of mutations that could inactivate a gene.

Using a forward mutation assay based on trimethoprim resistance, Sankar and colleagues comprehensively identified mutation signatures of conflicts [7]. Among a wide spectrum of loss-of-function mutations that were obtained, insertions/deletions (indels) and base substitutions at the promoter were demonstrated as the major mutation signatures resulting from replication-transcription conflicts. Indels were proximal to the sites where collisions occurred and spanned from *cis*-regulatory elements to coding regions. The vast majority of indels are deleterious, resulting in loss of gene function. Therefore, while there is no evidence that conflicts primarily generate beneficial mutations, there is conclusive evidence that conflicts generate deleterious mutations.

The forward mutation assay also detected hundreds of different base substitutions throughout the coding region and at the promoter. Mutation rate in the coding sequence (CDS) did not increase when head-on transcription was induced compared to the uninduced condition [7]. Therefore, most base substitutions in the CDS likely arise independently of head-on conflict. In contrast, promoter substitutions appeared as a hotspot with a strongly elevated rate upon induction of head-on transcription [7]. This hotspot is identical in nature and position to a promoter hotspot previously identified in a head-on oriented mutation reporter in *E*. *coli* [38]. This striking similarity in mutagenic signature identified in two phylogenetically distant bacteria (*E*. *coli* and *B*. *subtilis*), using two different reporter systems, supports the widespread existence of promoter substitutions due to head-on replication-transcription conflict.

Promoter substitutions due to head-on conflict suggest another origin of gene strand bias. A comparative genomic analysis demonstrated that long bacterial operons are strongly biased to the leading strand and occur sparsely in the head-on orientation, especially when the operons are conserved [39]. A single promoter mutation due to head-on conflict could inactivate every gene in a multigene operon, causing a strong loss of fitness. Therefore, promoter mutations caused by head-on conflicts are more likely to be deleterious than beneficial and thus convincingly explain the evolutionary pressure against head-on genes, especially for essential and highly expressed genes.

Recently, Lang and Merrikh argued against mechanistic evidence bolstering the evolutionary model of selection against head-on genes [15]. In their review, the authors conclude, mistakenly in our opinion, that promoter base substitutions due to head-on conflicts are a consequence of mutational bias introduced by toxic selection on trimethoprim [15]. We disagree with this interpretation for several reasons. First, the fundamental tenet of the fluctuation test as devised by Luria and Delbrück is that selection for mutants is separate from spontaneous generation of mutations during growth [1,2]. In the Luria and Delbrück experiment, phage-resistant mutant cells arose prior to exposure to phage, and not in response to phage infection [1]. Likewise, mutations resulting from replication-transcription conflicts were revealed by trimethoprim selection and were not generated in response to the drug, as demonstrated by the Luria-Delbrück distribution of mutants per culture [7]. Hence, mutations identified after selection for trimethoprim resistance represent authentic signatures of replication-transcription conflicts.

Second, if the argument is that promoter mutations negate the toxic nature of trimethoprim, then the same mutation should appear similarly enriched in the co-directional orientation, which was distinctly not the case. Additionally, the promoter mutant showed no growth advantage in competition with wild-type and other types of mutants, suggesting that it was a bona fide mutational outcome of conflicts and not a result of selection bias [7]. Therefore, loss-of-function mutation assays facilitate less biased estimation of mutation rates than reversion assays and are suitable for studying the impact of replication-transcription conflict on mutagenesis.

## Are mutations in head-on genes largely dependent on replication-transcription conflict?

Replication-transcription conflict is not the only mutagenic mechanism that can manifest in different mutation rates between co-directional and head-on genes. One such mechanism is differences between the accuracy of replication of the leading and lagging strands [40,41]. It is hypothesized that leading and lagging strand replication fidelity is unequal due to intrinsic differences in replication accuracy and error correction between leading and lagging strands during DNA replication, not necessarily due to replication-transcription conflict [40–43]. When a co-directional gene is inverted, its leading strand template would become the lagging strand template and vice versa. This would have the effect of adjusting mutation rate in the inverted gene due to changes in local genome context and error correction efficiency that are independent of conflict.

What about the mutation rate in genomes with an established equilibrium of head-on and co-directional genes? These questions can be addressed by mutation accumulation studies, which bypass the limitations of mutation reporters by using whole genome resequencing to identify mutations that become fixed in a population when selection is minimized. Many research groups have performed mutation accumulation experiments over the past decade and made key findings in the evolution of mutation rates, mechanisms of GC skew, the effect of replication timing on mutagenesis, and factors associated with spontaneous mutagenesis [22,23,37,44–49]. Strikingly, Foster, Lynch, and colleagues demonstrated that mutation accumulation studies have not found an association between base substitution rate and a gene’s orientation relative to replication [23]. Instead, one of the strongest determinants of substitution rate was the neighboring DNA sequence context at a given genomic locus [22,45]. Importantly, the influence of neighboring nucleotide sequence context on mutagenesis operates independently of the orientation and expression of a gene. Therefore, mutation accumulation studies suggest that in bacterial genomes that have established an equilibrium of head-on and co-directional genes, replication-transcription conflict is unlikely to be a major driver of mutagenesis.

## Are stress-induced genes typically head-on?

Mechanistic and evolutionary evidence clearly demonstrates that the majority of head-on oriented genes are not driven to be head-on through selection for conflict-induced adaptive mutagenesis. However, there is precedence for selection causing heritable genetic change. For example, upon phage infection, CRISPR loci acquire phage DNA in the form of new spacers [50,51]. Therefore, it remains possible that specific genes are head-on to benefit from conflict-induced mutability. Merrikh and colleagues proposed that stress-induced genes benefit from mutagenesis caused by head-on conflict when their expression is induced during stress [52]. We performed several analyses to test their model.

First, we examined the CDSs regulated by nine stress-associated transcription factors (Table 1). Are these stress-induced CDSs more likely to be oriented head-on, as the adaptive mutagenesis model for head-on genes suggests? No. On the contrary, all regulons except the SigV regulon contain more co-directional CDSs than head-on. For example, Paul and colleagues suggested that genes in the SigM (cell envelope stress) regulon were preferentially in the head-on orientation [19]. However, we found that there are 70 co-directional CDSs in the SigM regulon and 23 head-on. Furthermore, the Spx (oxidative stress) and Spo0A (sporulation) regulons were enriched for co-directional CDSs (Table 1). We conclude that, in stark contrast to the prediction of the adaptive hypothesis for head-on stress-induced genes, there is no compelling evidence for selection driving stress-induced genes to be head-on.

**Table 1:** Most stress-induced genes are co-directional.

Stress-associated regulator	Number of head-on CDSs in regulon (% of 1,177 total head-on)		Number of co-directional CDSs in regulon (% of 3,148 total co-directional)		*p*-Value (chi-squared)
LexA	14	(1.2%)	46	(1.5%)	0.59
SigB	72	(6.1%)	149	(4.7%)	0.078
SigM	23	(2.0%)	70	(2.2%)	0.67
SigV	14	(1.2%)	7	(0.22%)	0.00013
SigX	9	(0.07%)	33	(1.0%)	0.50
SigY	0	(0%)	7	(0.22%)	0.23
SinR	13	(1.1%)	36	(1.1%)	0.96
Spo0A	25	(2.1%)	117	(3.7%)	0.012
Spx*	3	(0.25%)	38	(1.2%)	0.0069

*Only CDSs activated by Spx are included.

The number of head-on and co-directional CDSs in *B*. *subtilis* strain 168 regulated by each transcription factor was tabulated. In addition, their percentage over the total number of head-on or co-directional CDSs is in parentheses.

Abbreviations: CDS, coding sequence; LexA, repressor of the SOS response to DNA damage; SigB, general stress response; SigM, cell envelope stress response; SigV, cell envelope stress response; SigX, cell envelope stress response; SigY, cell envelope stress response; SinR, biofilm; Spo0A, sporulation; Spx, oxidative stress

**Fig 2:**
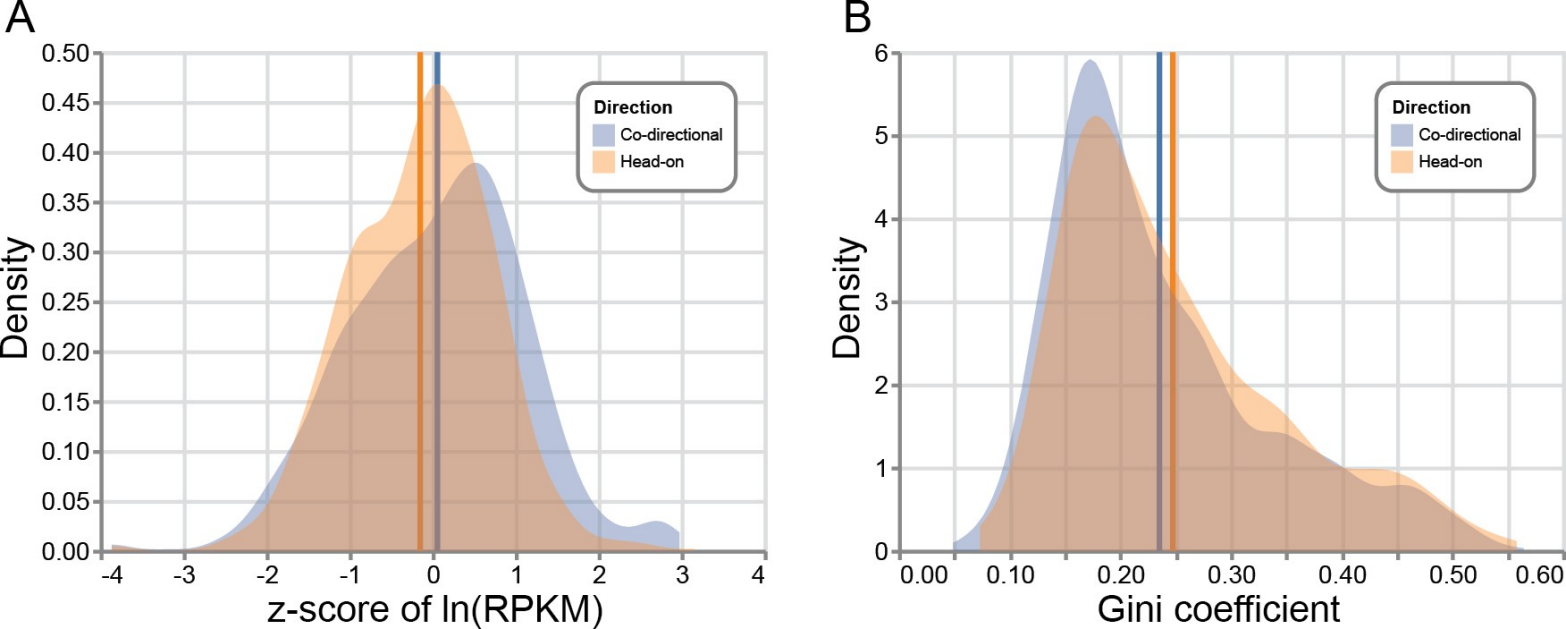
Head-on and co-directional genes have similar expression properties. Distributions of two metrics relating to gene expression in *B*. *subtilis* transcriptomic datasets. Distributions were subset by direction of transcription relative to DNA replication. Vertical lines represent the mean for each class of genes. (A) The distribution of gene expression values for CDSs in *B*. *subtilis* strain PY79. The z-score was calculated for each CDS’s natural-logarithm-transformed RPKM values prior to subsetting by direction. RNA-seq data are from [22]. (B) Transcriptomic microarray data [53] were analyzed. Each gene’s Gini coefficient was calculated over 104 conditions. CDS, coding sequence; RPKM, reads per kilobase per million reads mapped

Second, under the assumption that stress-induced genes are preferentially head-on, Lang and Merrikh suggested that the reason mutation accumulation studies have not found head-on genes to have higher mutation rates than co-directional genes is due to the relatively low-stress conditions of mutation accumulation experiments [23]. However, RNA-seq data show that head-on CDSs occupy a similar range of expression values as co-directional CDSs (Fig 2A) [22]. To further test whether head-on genes tend to be “off” under most conditions and “on” under a select few, we analyzed *B*. *subtilis* transcriptomic data from [53], in which gene expression was measured under 104 conditions. We estimated each gene’s propensity for differential expression by calculating its Gini coefficient over the 104 conditions. The range of Gini coefficients was nearly identical between head-on and co-directional genes (Fig 2B). These analyses indicate that head-on genes are not particularly special in their patterns of differential expression when compared with co-directional genes.

## Conclusions

Genome organization has been shaped by selective forces that minimize replication-transcription conflict, resulting in enriched co-directionality between replication and transcription. Although the hypothesis that positive selection promotes head-on orientation of stress-induced genes to increase conflict and promote adaptive mutation is tempting [19], evolutionary [20,21,23] and mechanistic analyses [7,38] suggest this is not the case. Together, the mutation-selection balance theory and conflict-induced mutation signatures strongly support the evolutionary model that head-on genes are a result of inevitable gene inversion [20,31,36], to be later purified from the genome due to increased deleterious mutation rate and their greater propensity to block DNA replication [7,10,13,14,20,38]. Genes remaining head-on tend to be those in which purifying selection is relaxed. Moreover, the distribution of stress-induced genes is not significantly different between head-on and co-directional orientations. Hence, the adaptive evolution model of head-on genes is poorly suited to explain the presence of genes on the lagging strand and still requires compelling empirical support. Genomes in their current state have likely evolved to an equilibrium distribution of co-directional and head-on genes that is maintained by selective pressure against deleterious consequences resulting from genome rearrangements.

## Methods

Transcriptomic microarray data were downloaded from http://genome.jouy.inra.fr/basysbio/bsubtranscriptome. The intercept log_2_(signal) for each gene and the effects of all of the 104 conditions tested in [53] on each gene’s log_2_(signal) were inferred. To avoid obtaining many false positive effects, a Bayesian model with a shrinkage prior known as the Finnish Horseshoe prior [54] was employed.

Gene enrichment analysis was performed using regulon annotations hosted at the Subtiwiki database [55]. A chi-squared test for independence between gene regulation by each regulator and gene direction relative to DNA replication was used to calculate the *p*-values in Table 1.

A detailed description of our methods, including all R and Python code, and all the data required to reproduce our analyses, can be found in the github repository hosted at https://github.com/jadewanglab/2020_PGen_analysis.git.
